# Effect of different doses of Budesonide combined with Tiotropium bromide inhalation on elderly patients with chronic obstructive pulmonary disease

**DOI:** 10.12669/pjms.40.7.9209

**Published:** 2024-08

**Authors:** Jianhong Yu, Jianchao Ni, Xindong Chen, Yuanyuan Fang, Suna Fu

**Affiliations:** 1 Jianhong Yu, Department of Geriatric, Affiliated Hospital of Shaoxing University, 999 Zhongxing South Road, Shaoxing, Zhejiang Province 312000, P.R. China; 2 Jianchao Ni Department of Geriatric, Affiliated Hospital of Shaoxing University, 999 Zhongxing South Road, Shaoxing, Zhejiang Province 312000, P.R. China; 3 Xindong Chen Department of Geriatric, Affiliated Hospital of Shaoxing University, 999 Zhongxing South Road, Shaoxing, Zhejiang Province 312000, P.R. China; 4 Yuanyuan Fang Department of Geriatric, Affiliated Hospital of Shaoxing University, 999 Zhongxing South Road, Shaoxing, Zhejiang Province 312000, P.R. China; 5 Suna Fu Department of Geriatric, Affiliated Hospital of Shaoxing University, 999 Zhongxing South Road, Shaoxing, Zhejiang Province 312000, P.R. China

**Keywords:** Budesonide, Chronic obstructive pulmonary disease, Different doses, Tiotropium bromide

## Abstract

**Objective:**

To explore the clinical effect of various doses of Budesonide combined with Tiotropium bromide in the treatment of elderly patients with chronic obstructive pulmonary disease (COPD).

**Methods:**

Clinical data of elderly patients with COPD, admitted to Affiliated Hospital of Shaoxing University from April 2021 to February 2023, were retrospectively analyzed. Based on the dosage of Budesonide combined with Tiotropium bromide, patients were divided into Low-dose group (Budesonide = 1mg), Medium-dose group (Budesonide = 2mg), and High-dose group (Budesonide = 3mg). All groups were matched for age, gender, course of disease, and BMI. Patients treated with Tiotropium bromide alone were assigned to the Control group. The clinical effect, pulmonary function index level, symptom improvement, inflammatory factor index level and adverse reactions in all groups were analyzed and compared.

**Results:**

A total of 88 patients were included in this study with 22 patients in each group. The total efficacy of Medium-dose (90.91%) and High-dose group (90.91%) was significantly higher than that of Low-dose group (63.64%) and the Control group (59.09%) (*P*<0.05). After the treatment, levels of pulmonary function, symptom improvement and inflammatory factors in the High-dose and the Medium-dose groups were better than those in the Low-dose group and the Control group. Pulmonary function, symptom improvement and levels of inflammatory factors was significantly better in the Low-dose group compared to the Control group (*P*<0.05).

**Conclusions:**

Budesonide combined with tiotropium bromide is better than tiotropium bromide alone in the treatment of elderly patients with COPD. Compared with low (1mg) dosage, medium (2mg) and high (3mg) dosage of budesonide are more effective in improving lung function, alleviating symptoms, reducing inflammatory response,, and are not associated with increased rate of adverse reactions.

## INTRODUCTION

Chronic obstructive pulmonary disease (COPD) is the third leading cause of death worldwide, and is common in the elderly population.^1^,^2^ In the absence of timely and effective intervention, the disease may progress to cor pulmonale and respiratory failure, posing a great threat to the life and health of patients.^1^-^3^

Tiotropium bromide, which is commonly used in clinical treatment of COPD, is a long-acting anticholinergic drug that can selectively act on M1 and M3 receptors, inhibit continuous bronchoconstriction, and play an important role in reducing inflammatory response and improving lung function.^4^,^5^ Budesonide is a commonly used anti-inflammatory glucocorticoid that characterized by non-specific anti-inflammatory effect and a rapid onset.^6^chronic obstructive pulmonary disease (COPD Budesonide is a commonly used inhaled glucocorticoid for COPD, and is effective in efficiently alleviating clinical symptoms and improving lung function.^6^,^7^

Several studies have reported combined application of tiotropium bromide and glucocorticoids in patients with COPD, but with conflicting results.^8^-^10^ Additionally, the optimal dosage of budesonide when combining with Tiotropium bromide in treating patients with COPD in clinical practice is still unclear. This study retrospectively analyzed the clinical data of patients with COPD who received different doses of budesonide combined with tiotropium bromide to assess the efficiency of the combined treatment regimen and to determine the optimal dosage of budesonide for the treatment of COPD in elderly patients.

## METHODS

We retrospectively collected clinical data from 88 patients with COPD (54 males and 34 females) admitted to Affiliated Hospital of Shaoxing University from April 2021 to February 2023. A total of 22 patients were treated with 18μg of tiotropium bromide alone, and were set as the Control group; 22 patients received low-dose (1mg) budesonide combined with tiotropium bromide, and were set as the Low-dose group; 22 patients received medium dose (2mg) of budesonide combined with tiotropium bromide, and were set as the Medium-dose group; 22 patients received high dose (3mg) budesonide combined with tiotropium bromide, and were set as the High-dose group. All groups were matched for age, gender, course of disease, and BMI.

### Ethical Approval:

The ethics committee of Affiliated Hospital of Shaoxing University approved our study on November 12^th^, 2021. No.: 2021-042-02.

### Inclusion criteria:

- Patients met the diagnostic criteria for COPD.^11^
- Age ≥ 60 years old.
- The clinical data was complete.

### Exclusion criteria:

- Individuals with mental illness.
- Individuals with severe kidney, liver, and heart conditions.
- Patients allergic to tiotropium bromide and/or budesonide.
- Patients complicated with bronchial asthma, pneumothorax, or pulmonary embolism.
- Patients who received hormones and bronchiectasis agents within four weeks before being included in the study.
- Autoimmune diseases.

### Treatment methods:

All patients were given treatments such as reducing phlegm, relieving cough, relieving asthma, inhaling oxygen, and correcting electrolyte and acid-base imbalance. All patients received tiotropium bromide (Jiangsu Zhengda Tianqing Pharmaceutical Co., Ltd., Jiangsu, China; specification: 18μg per capsule) inhaled 18μg/time, once a day. Patients who received the combined treatment also received aerosol inhalation of budesonide suspension (AstraZeneca Pty Ltd., Sydney, Australia; specification: 2ml: 0.5mg) 1~3mg (1mg, 2mg, and 3mg for low dose group, medium lose group, and high dose group, respectively) 20minutes/time, one time/day. The treatment was continued for two weeks.

### Outcome measures:

***Clinical effect:*** -

- ***Obviously effective:*** The complete disappearance of pulmonary rales or the presence of some dry rales, significant improvement in lung function, and the disappearance or significant improvement of clinical symptoms. –
- ***Effective:*** Improvement in lung function, but with some shadowing, the significant reduction of dry rales in the lungs, and the alleviation of symptoms such as shortness of breath, cough and sputum.-
- ***Ineffective:*** No evidence of symptom alleviation. Obviously Effective and Effective were included in the total effective score.^12^

### Lung function indicators:

The levels of forced expiratory volume in the first second (FEV1) and FEV1 to maximum vital capacity ratio (FEV1/FVC) were included. Lung function indicators were measured using the MS-iOS lung function detector (JAEGE company, Germany).

### Symptom improvement:

Improvement was evaluated based on the Modified Respiratory Difficulty Index (mMRC) and COPD Assessment Test Score (CAT).^13^ Briefly, mMRC scores 0, one, two, three and four based on levels zero, one, two, three and four respectively, with lower the score indicating better outcome. CAT score assesses breathing difficulties, coughing, confidence in outdoor activities, expectoration, sleep, chest tightness, daily life limitations, and mental state, with a total score of five points for each item, totaling 40 points. Lower score is associated with better improvement.

### Inflammatory factor indicators:

Levels of high sensitive C-reactive protein (hs-CRP), tumor necrosis factor-α (TNF-α) and Interleukin 8 (IL-8) were measured in the serum of 4 ml of blood by enzyme-linked immunosorbent assay. The reagent kit was purchased from Wuhan Doctoral Biotechnology Co., Ltd. 5)

### Adverse reactions:

Adverse reactions such as diarrhea, rash, and nausea were recorded.

### Statistical Analysis:

The statistical software used was SPSS22.0. The measurement data, meeting normal distribution, were expressed by (*χ̅*±*S*). Multiple group comparisons were conducted using one-way ANOVA, and pairwise comparisons were conducted using LSD test afterwards. Paired t-tests were used for intragroup comparison before and after the treatment. The measurement data that did not meet normal distribution were represented by M (IQR). Kruskal wails *H* test was used for intergroup comparison, and the Nemenyi method was used for pairwise comparison. The Wilcoxon signed rank test was used for intragroup comparison before and after the treatment. Counting data were expressed by n (%), and were compared by Chi-squared test or Fisher’s exact test. According to the test level, *P*<0.05 was considered statistically significant.

## RESULTS

A total of 88 patients met the requirements for this study. The age of the patients ranged from 60 to 83 years, with an average of 69.99 ± 5.59 years. The course of the disease was 1-11 years, with an average of 5.47 ± 2.04 years.. There was no significant difference in the baseline data among the four groups (*P*>0.05), Table-I.

**Table-I:** Comparison of baseline data for four groups.

Group	n	Gender (Male/female)	Age (years)	Course of disease (Year)	BMI (kg/m^2^)
High-dose group	22	15/7	69.95±6.48	5.68±2.30	23.69±2.54
Medium-dose group	22	12/10	71.59±5.96	4.91±1.95	23.01±2.78
Low-dose group	22	14/8	68.41±5.41	5.77±1.85	24.06±2.19
Control group	22	13/9	70.00±4.20	5.50±2.06	24.15±2.18
*χ^2^/F*		0.959	1.194	0.791	0.997
*P*		0.811	0.317	0.502	0.398

The total efficacy of the Medium-dose group (90.91%) and the High-dose group (90.91%) was significantly higher than that of the Low-dose (63.64%) and the Control groups (59.09%) (*P*<0.05). There was no significant difference in the total efficacy between the High-dose and the Medium-dose groups, and between the Low-dose group and the Control group (*P*>0.05), Table-II.

**Table-II:** Comparison of treatment effects among four groups.

Group	n	Significant effect	Effective	Invalid	Total effective rate
High-dose group	22	14 (63.64)	6 (27.27)	2 (9.09)	20 (90.91)^a^
Medium-dose group	22	12 (54.55)	8 (36.36)	2 (9.09)	20 (90.91)^a^
Low-dose group	22	9 (40.91)	5 (22.73)	8 (36.36)	14 (63.64)
Control group	22	7 (31.82)	6 (27.27)	9 (40.91)	13 (59.09)

***Note:*** Compared with low-dose group and Control group,

a P<0.05.

Before the treatment, levels of FEV1, FEV1/FVC were comparable in the four groups (*P*>0.05). After the treatment, the levels of FEV1, FEV1/FVC in the four groups increased compared to the pretreatment levels (*P*<0.05). Levels of FEV1 and FEV1/FVC in the High-dose, Medium-dose and Low-dose groups were significantly higher than those in the Control group (*P*<0.05). FEV1 and FEV1/FVC values in the High-dose and Medium-dose groups were significantly higher than those in the Low-dose group (*P*<0.05). There was no significant difference between the High-dose group and the Medium-dose group (*P*>0.05), Table-III.

**Table-III:** Comparison of lung function among four groups.

Time	Group	n	FEV1 (L)	FEV1/FVC (%)
Before treatment	High-dose group	22	1.20±0.08	56.30±7.43
	Medium-dose group	22	1.18±0.10	55.59±7.07
	Low-dose group	22	1.22±0.09	56.98±6.94
	Control group	22	1.20±0.14	55.56±4.71
	F		0.353	0.227
	P		0.787	0.877
After treatment	High-dose group	22	1.90±0.33^ab*^	69.71±7.02^ab*^
	Medium-dose group	22	1.89±0.28^ab*^	69.00±5.98^ab*^
	Low-dose group	22	1.72±0.26^a*^	64.83±3.39^a*^
	Control group	22	1.52±0.23^*^	61.41±3.98^*^
	F		9.365	11.742
	P		<0.001	<0.001

***Note:*** Compared with the Control group,

a P<0.05; Compared with the low-dose group, ^b^P<0.05; Compared with before treatment,

* P<0.05.

Before the treatment, there was no significant difference in the mMRC and CAT scores in all groups (*P*>0.05) After the treatment, the mMRC and CAT scores of the four groups decreased compared to pretreatment levels (*P*<0.05), and were significantly lower in three groups of patients who received the combined treatment compared to the Control group of patients (tiotropium bromide alone). Among the patients who were given the combined treatment, high and medium doses of budesonide were associated with significantly lower mMRC and CAT scores than those of the Low-dose group (*P*<0.05). There was no significant difference between the High-dose group and the Medium-dose group (*P*>0.05), Table-IV.

**Table-IV:** Comparison of symptom improvement among four groups (score).

Time	Group	n	mMRC	CAT
Before treatment	High-dose group	22	3(3,4)	16(14,17)
	Medium-dose group	22	3(3,3)	16(15,17)
	Low-dose group	22	3(3,4)	16(16,17)
	Control group	22	3(3,3)	16(15,17)
	H		0.165	0.682
	P		0.983	0.877
After treatment	High-dose group	22	2(2,2)^ab*^	8(7,9)^ab*^
	Medium-dose group	22	2(2,2)^ab*^	8.5(7,9)^ab*^
	Low-dose group	22	2(2,3)^a*^	10(9,11)^a*^
	Control group	22	3(2,3)^*^	11(10,12)^*^
	H		27.559	54.655
	P		<0.001	<0.001

***Note:*** Compared with the Control group,

a P<0.05; Compared with the low-dose group, ^b^P<0.05; Compared with before treatment,

* P<0.05.

Before the treatment, there was no significant difference in the levels of hs-CRP, TNF-α, and IL-8 among the four groups (*P*>0.05). After the treatment, the serum levels of hs-CRP, TNF-α, and IL-8 in all groups significantly decreased (*P*<0.05), and were significantly lower the High-dose, the Medium-dose, and the Low-dose group compared to the Control group. The average levels of hs-CRP, TNF -, and IL-8 in the High-dose and Medium-dose groups were significantly lower than those in the Low-dose group (*P*<0.05). However, there was no significant difference between the High-dose and Medium-dose groups (*P*>0.05), Table-V.

**Table-V:** Comparison of levels of inflammatory factor indicators among four groups.

Time	Group	n	hs-CRP (mg/L)	TNF-α (ng/L)	IL-8 (ng/L)
Before treatment	High-dose group	22	63.63±7.19	39.19±6.36	30.66±6.18
	Medium-dose group	22	65.09±6.29	40.51±6.17	31.18±5.80
	Low-dose group	22	64.41±8.65	40.94±5.21	30.80±4.74
	Control group	22	65.08±8.84	39.50±5.85	31.49±6.22
	F		0.171	0.430	0.093
	P		0.915	0.732	0.964
After treatment	Group	22	11.47±4.61^ab*^	12.30±3.76^ab*^	9.54±4.10^ab*^
	High-dose group	22	12.12±2.98^ab*^	13.54±4.46^ab*^	10.00±4.11^ab*^
	Medium-dose group	22	16.70±5.30^a*^	17.14±5.34^a*^	14.08±4.00^a*^
	Low-dose group	22	19.46±3.93^*^	20.10±4.57^*^	17.78±4.12^*^
	F		17.290	13.226	19.74
	P		<0.001	<0.001	<0.001

***Note:*** Compared with the Control group,

a P<0.05; Compared with the low-dose group, ^b^P<0.05; Compared with before treatment,

* P<0.05.

The incidence of adverse reactions was 9.09% in the High-dose group and the Medium-dose group, 4.55% in the Low-dose group, and 4.55% in the Control group, comparable in all four groups (*P*>0.05), Table-VI.

**Table-VI:** Comparison of adverse reactions among four groups.

Group	n	Diarrhea	Rash	Nausea	Total occurrence rate
High-dose group	22	1 (4.55)	0 (0.00)	1 (4.55)	2 (9.09)
Medium-dose group	22	0 (0.00)	1 (4.55)	1 (4.55)	2 (9.09)
Low-dose group	22	0 (0.00)	0 (0.00)	1 (4.55)	1 (4.55)
Control group	22	1 (4.55)	0 (0.00)	0 (0.00)	1 (4.55)
Fisher’s exact test					1.544
P					0.736

## DISCUSSION

The results of this study showed that budesonide combined with tiotropium bromide was significantly more effective than tiotropium bromide alone in improving lung function, inflammatory factor levels and alleviating symptoms of patients with COPD. There was no significant difference in the rate of adverse reactions between two treatment regimens. Our results confirm that budesonide combined with tiotropium bromide is helpful to further improve the disease treatment effect, reduce clinical symptoms and inflammatory reactions in patients with COPD, and effectively restores their lung function. The administration of medium and high doses of budesonide can improve the overall clinical effect of the treatment better than small doses, and the increase of drug dosage is not associated with an increased risk of adverse reactions.^14^,^15^

Hanania et al.^14^ used high-dose and low-dose budesonide to treat patients with COPD, and showed that the improvement of lung function and acute attack in patients treated with high dose of the drug were better compared to the low-dose group. The incidence of adverse reactions in both groups was low, which indicates the safety of high-dose budesonide. Bagherisadeghi et al.^15^ also confirmed that increasing the dosage of budesonide has certain positive impact on improving the pulmonary function rehabilitation effect of patients with COPD, and does not correlate with higher incidence of adverse reactions. Qi et al.^16^ showed that the optimal dose of budesonide for patients with COPD in China is 4-6 mg/d. Meta-analysis by Li et al.^17^ also confirmed that compared with the conventional dose of budesonide, high-dose drug regimen can more effectively regulate the arterial blood gas status of patients with COPD, alleviate clinical symptoms, and help restore the lung function of patients. Our study is consistent with the above research results.However, their research also showed that high-dose budesonide may increase the risk of oral fungal infection. Zhang et al.^18^ used low-dose, medium dose and high-dose budesonide to treat patients with COPD on the basis of salbutamol sulfate, and showed that increased dosage of budesonide was associated with improved treatment effect. Higher doses of budesonide were more efficient in improving patient’s lung function, lowering the expression of inflammatory factors such as hs-CRP, alleviating the degree of inflammatory response, and ensuring overall safety. Follow-up studies need to further explore the optimal dose of budesonide in the treatment of COPD.

Tiotropium bromide, an anticholinergic agent commonly used in COPD can specifically bind to the muscarine receptor in the bronchial smooth muscle cells to inhibit the production of acetylcholine.^19^ This can relieve the contraction of bronchial smooth muscle cells and improve the status of bronchospasm. However, it is difficult to achieve the expected clinical level of the overall effect just with tiotropium bromide alone. Therefore, a combined treatment regimen is necessary.^19^,^20^ Budesonide is an important glucocorticoid in the clinical treatment of COPD, with significant anti-inflammatory effect. 21 It can inhibit the production of various inflammatory factors mediated by neutrophils, and reduce the edema of small airways in lung tissue and respiratory epithelium.^21^,^22^

Similar to our result, Usmani O et al.^23^ also pointed out that budesonide is also effective in controlling the inflammatory reaction of small airway and respiratory tract, and downregulates the expression of neutrophils and leukocytes and downstream production of chemokines and interleukins by monocytes and neutrophils. Together, these may help to control the downstream inflammatory reaction. While current studies show clinical value of budesonide combined with tiotropium bromide in the treatment of COPD,^24^ there is no unified standard for the optimal dosage of Budesonide.^15^,^16^ Our study clearly shows that medium and high doses of Budesonide can improve disease treatment effect and ensure treatment safety. We may speculate that as the dosage of the medication increases, the local drug concentration rises, making it more effective in targeting the lesion and continuously exerting its efficacy. At the same time, the form of nebulization inhalation not only ensures that the drug directly reaches the lesion, but also does not cause systemic adverse reactions, thus ensuring the safety of the treatment.

### Limitations:

This is a single center retrospective analysis, with a small sample size and selection bias. There are few observation indicators and a lack of more comprehensive evaluation of treatment effectiveness. Additionally, there is currently no clinical cure for COPD, and the clinical treatment of this disease is a long process. So the lack of long-term follow-up and failure to evaluate long-term efficacy after treatment is also a limitation of the study. Further studies are needed to address these limitations and further explore the optimal dose of budesonide in the treatment of COPD in elderly patients.

## CONCLUSION

Budesonide combined with tiotropium bromide is more effective than tiotropium bromide alone in the treatment of elderly patients with COPD. Compared with low (1mg) dosage, medium (2mg) and high (3mg) dosage of budesonide are more effective in improving lung function, alleviating symptoms, reducing inflammatory response, and not associated with increased risk of adverse reactions.

### Authors’ contributions:

**JY:** Conceived and designed the study.

**JN**, **XC**, **YF** and **SF:** Collected the data and performed the analysis.

**JY:** Was involved in the writing of the manuscript and is responsible for the integrity of the study.

All authors have read and approved the final manuscript.
